# Oncostatin M stimulates cell migration and proliferation by down-regulating E-cadherin in HTR8/SVneo cell line through STAT3 activation

**DOI:** 10.1186/1477-7827-11-93

**Published:** 2013-09-24

**Authors:** Hyun Sun Ko, Sae Kyung Choi, Hee Kyung Kang, Ho Shik Kim, Ji Hyun Jeon, In Yang Park, Jong Chul Shin

**Affiliations:** 1 Department of Obstetrics and Gynecology, College of Medicine, Catholic University, Seoul, Korea; 2 Department of Biochemistry, College of Medicine, Catholic University, Seoul, Korea; 3 Department of Anatomy, College of Medicine, Catholic University, Seoul, Korea

**Keywords:** Oncostatin M (OSM), Trophoblast, Migration, Proliferation, E-cadherin, Signal transducer and activator of transcription (STAT)3

## Abstract

**Background:**

During the first trimester of pregnancy, trophoblastic E-cadherin expression is down-regulated, thereby allowing extravillous trophoblasts (EVTs) to acquire the potential for migration and invasiveness. The aim of the present study was to investigate the role of OSM on the migration and proliferation of EVT cell line HTR8/SVneo with regard to its effects on the expression of E-cadherin and STAT3 activation.

**Methods:**

We investigated the effects of OSM on RNA and protein expression of E-cadherin by real time RT-PCR analyses, western blotting, and indirect immunofluorescence staining in HTR8/SVneo cells, as well as the effects on cell migration and proliferation. The selective signal transducer and activator of transcription (STAT)3 inhibitor, stattic, and STAT3 siRNA were used to investigate STAT3 activation by OSM.

**Results:**

OSM significantly reduced RNA and protein expression of E-cadherin. Indirect immunofluorescence staining of HTR8/SVneo cells also revealed the down-regulation of E-cadherin, compared with the controls. OSM-stimulated cell migration was attenuated by anti-gp130 antibodies. OSM-induced STAT3 phosphorylation, and the down-regulation of E-cadherin by OSM treatment was restored by stattic and STAT3 siRNA. In addition, OSM-stimulated migration and proliferation were significantly suppressed by STAT3 inhibition.

**Conclusions:**

This study suggests that OSM stimulates the migration and proliferation of EVTs during the first trimester of pregnancy through the down-regulation of E-cadherin. In addition, this study suggests that the effects of OSM on migration and proliferation are related to STAT3 activation, which is important in trophoblast invasiveness.

## Background

In early pregnancy, extravillous trophoblasts (EVTs) invade through the endometrium, interact with decidual and immunocompetent cells, and differentiate into multinucleated placental bed giant cells. In addition, they can invade the maternal spiral arteries, mediate the destruction of the arterial wall, and replace the endothelium by forming endovascular trophoblasts [1,2].

During early pregnancy, the invasion of human trophoblast cells into the uterus is one of the essential events for the establishment of a successful pregnancy. It has been proposed that the processes by which placental cytotrophoblast cells change phenotypes from being coherently attached to being migratory, where cells invade the maternal decidua, resemble other developmental epithelial mesenchymal transitions (EMTs) [3-5]. Because this transition is critical in normal placental development, growth, migration, and invasion, it raises the question as to which factors regulate these migratory events and how the altered regulation of this transition might manifest pathologically. Given the importance of the modulation of cell-cell adhesions in EMTs, investigation of the factors that regulate cell adhesion and invasion in the placenta might lead to the further understanding of the early events surrounding placental development in normal and pathological pregnancies. The modulation of cell adhesion and cell polarity occurs through changes in cell-cell junctional molecules such as cadherins. Cadherins, particularly the classical cadherins (e.g., epithelial (E)-cadherin), and their linkage to adaptors called catenins, at cell-to-cell contacts, are important for maintaining cell attachment and the layered phenotype of villous cytotrophoblasts. In contrast, the reduced expression and re-organization of cadherins from these cell junctional regions promotes the loosening of connections between cells and reduced apico-basal polarity.

Oncostatin M(OSM) is a member of the interleukin (IL)-6 family of cytokines, which includes IL-6, leukemia inhibitory factor (LIF), ciliary neurotrophic factor, cardiotrophin-1, IL-31, and IL-11. OSM is a pleiotropic cytokine secreted by neutrophils, macrophages, and activated T cells. OSM is known to be elevated in patients with rheumatoid arthritis and chronic periodontal disease, and it plays a significant role in the inflammatory process [6]. In addition, OSM can act as either a stimulator or an inhibitor in the regulation of proliferation and differentiation.

Little is known about the effects of OSM on pregnancy, although OSM concentrations in the sera of pregnant women were found to be significantly higher than that in the sera of non-pregnant women, throughout the pregnancy period [7,8]. It is possible that OSM may affect the invasion and migration processes of the EVTs through various mechanisms, including its effect on EMT during early pregnancy. Our previous *in vitro* study demonstrated that OSM increases the invasion of EVTs in a first trimester EVT cell line [9]. It has been reported that the loss of E-cadherin with an increase of snail, which represses the transcription of E-cadherin, is accompanied with an EMT in trophoblasts [10]. The aim of the present study was to investigate the role of OSM on EVT migration and proliferation with regard to its effects on the expression of E-cadherin, as a negative regulator of invasive behavior and related signaling pathways.

## Methods

### Cell lines

The EVT cell line HTR8/SVneo was kindly provided by Dr. Charles Graham (Queen’s University, Kingston, ON, Canada). The cell line was produced by immortalization of HTR8 cells, an EVT cell line from primary explant cultures of first-trimester human placenta (8- to 10-week gestation), with SV40 [11]. These cells exhibit markers of primary EVT cells, including the cytokeratins KRT7, KRT8, and KRT18, placental-type alkaline phosphatase, high-affinity PLAUR, human leukocyte antigen (HLA) framework antigen W6/32, HLA-G, insulin-like growth factor 2 (IGF2) mRNA, and a selective repertoire of integrins such as ITGA1, ITGA3, ITGA5, ITGAV, ITGB1, and ITGAVB3/B5 [12,13]. In the present study, HTR8/SVneo cells were used between passages 70 and 75.

### Cell culture

HTR8/SVneo cells were cultured in RPMI1640 (GIBCO, Grand Island, NY, USA) containing 10% FBS. To analyze the effects of OSM on E-cadherin in HTR8/SVneo cells, 10^7^ cells were seeded in a 100-mm culture dish. After 24 h, the cells were treated with recombinant human OSM (20 ng/mL; Sigma-Aldrich, St. Louis, MO, USA) for the time indicated in the figure legends.

### Real-time quantitative RT-PCR analysis

Total RNA was extracted with TRIZOL reagent (GIBCO-BRL, Int). The sequences of the primers used for real-time PCR analysis for E-cadherin (GenBank Accession No. NM_004360.3) and GAPDH (used as an internal control) were as follows: E-cadherin (Forward 5′-CGC GTC CTG GGC AGA GTG AAT TTT G-3′); GAPDH (Forward, 5′-CGG AGT CAA CGG ATT TGG TCG TAT-3′) [14].

#### cDNA synthesis

cDNA was synthesized with 500 ng of RNA using the Superscript™ ∥ RT-PCR System (Invitrogen, Karlsruhe, Germany) according to the manufacture’s recommendations. cDNA was diluted 1:2 prior to use in quantitative PCR.

#### Quantitative TaqMan PCR

PCR was performed in an ABI PRISM 7900HT Sequence Detection System (Applied Biosystems, Foster City, CA, USA) in 384-well microtiter plates, with a final volume of 10 μL. Optimum reaction conditions were established by using 5 μl of Universal Master Mix (Applied Biosystems, Foster City, CA, USA) containing dNTPs, MgCl2, reaction buffer and Ampli Taq Gold, 90 nM of primer(s) and 250 nM fluorescence-labeled TaqMan probe. Finally, 2 μl template cDNA was added to the reaction mixture. The primer/TaqMan probe combinations were designed for each target sequence. The assay ID for the E-cadherin probe was Hs01023894_m1 (Life technology). The thermal cycling conditions used were as follows: an initial DNA denaturation step at 95°C for 10 min, followed by 40 cycles of denaturation at 95°C for 15 s, primer annealing at 60°C for 1 min, and an extension step at 72°C for 15 s. All samples were amplified in triplicate, and data were analyzed with Sequence Detector software (Applied Biosystems).

### Western blot analysis

The HTR8/SVneo cells were seeded in 6-well cell culture plates (0.1 × 10^6^ cells per well) in RPMI-1640 medium supplemented with 10% FBS and cultured to 70–80% confluency. The cells were incubated for 48 h, with or without OSM (20 ng/mL). After incubation, the cells were washed with Dulbecco’s Phosphate-Buffered Saline (DPBS), and protein was extracted using RIPA lysis and extraction buffer (Pierce, USA). Next, 1 mL of extracted protein was centrifuged at 12,000 rpm for 10 min to remove the residual cell sediment and was quantified using BCA protein assay reagent (Pierce, USA). Then, 50 μg of protein were mixed with 5× sample loading buffer (Fermentas, Canada) and denatured at 100°C for 5 min. The mixture was then subjected to electrophoresis on an 8–16% SDS-PAGE gel (Koma Biotech, Korea) at 125 V for 2.5 h and then transferred to a nitrocellulose membrane (Genescript, USA). We used GAPDH (Santa Cruz, USA) as a loading control. After the transfer, the membrane was blocked for 1 h with Noise-Cancelling Reagents (Millipore, USA) and then incubated overnight at 4°C with a mouse anti-human E-cadherin (1:100) (Santa Cruz, USA). Membranes were rinsed in 10 mM Tris, 150 mM NaCl (TBS) and 0.1% Tween 20 prior to, and after incubation with horseradish peroxidase-conjugated anti-mouse IgG (ICN Biomedicals, Aurora, OH). Chemi-luminescence was detected with Luminata Crescendo Western HRP substrate (Millipore, USA) and autoradiography film (Agfa, Belgium) according to the manufacturer’s instructions. The experiment was replicated 3 times. The western blot bands were quantified by Gel Doc™ XR + with Image lab software (Bio Rad, USA).

#### Signal transducer and activator of transcription (STAT)3 phosphorylation by OSM

The HTR8/SVneo cells (0.1 × 10^6^ cells per well) were seeded in 6-well cell culture plates in RPMI-1640 medium supplemented with 10% FBS and cultured until 70–80% confluency was reached. The cells were treated with OSM (20 ng/mL) for 5 min, 15 min, 30 min, 1 h, 3 h, or 8 h. The control cells were incubated for 8 h without OSM. The western blot protocol was the same as that described above except that the antibodies used were as follows: mouse anti-human phosphorylated (phosphorylation of Tyr705) STAT3 (1:200) and mouse anti-human total STAT3 (1:50) (Santa Cruz, USA). The effect of OSM on STAT3 phosphorylation was examined following pretreatment with 1 μM stattic for 1 h (Tocris, R&D system).

#### The effect of STAT3 inhibition on OSM-mediated changes in E-cadherin in HTR8/SVneo cells

HTR8/SVneo cells (0.1 × 10^6^ cells per well) were seeded in 6-well cell culture plates in RPMI-1640 medium supplemented with 10% FBS and cultured until 70–80% confluency was reached. The cells were treated with OSM (20 ng/mL) for 48 h with or without stattic (0.5 μM or 1 μM for 1 h) pretreatment prior to western blotting. The subsequent steps were the same as described above.

##### STAT3 siRNA and transfections

The double-stranded siRNA oligonucleotide against STAT3 has the sequence 5′-AATGTTCTCTATCAGCACAAT-3′ [15]. Oligonucleotides were synthesized by Genolution Pharmaceuticals, Inc. (Seoul, Korea). Negative controls consisted of a well-tested non-targeting scrambled siRNA with no homology to mammalian genes. HTR-8/SVneo cells were seeded in 6-well plates just prior to transfection. For optimum transfection efficacy, cells were seeded to a final cell confluency of 30–50%. Cells were transfected with either STAT3 siRNA (25 nM) or scrambled siRNA (25 nM) complexed with G-Fectin (Genolution Pharmaceuticals, Inc., Seoul, Korea) for 24 h. After treatment with OSM (20 ng/mL) for 48 h, cells were dislodged from the surface of 6-well culture plate for western blotting.

### Indirect immunofluorescence

Cells were cultured on microscope cover slips (diameter, 12 mm; Marienfeld GmbH & KG, Germany). Thereafter, the cells were stimulated with 20 ng/mL OSM or left untreated for 48 h, with or without stattic (1 μM for 1 h) pretreatment, and then fixed with 4% paraformaldehyde (Wako, Japan) in 0.01 M phosphate-buffered saline (PBS pH 7.4) for 5 min at room temperature. Next, these cells were incubated in 2% BSA containing 0.1% Triton X-100 for 30 min at room temperature. Triton was used for permeabilization. We tested several blocking methods and solutions and found that 2% BSA was ideal as a blocking solution. Cells were then incubated with a mouse anti-human monoclonal antibody against E-cadherin (1:100; BD Transduction Laboratories) in blocking solution for 1 day at 4°C, to allow good penetration of the primary antibodies. The cells were washed in PBS and incubated in the presence of appropriate secondary antibodies conjugated with Cy3 (1:1000; Jackson Immuno Research) for 2 h at room temperature. The fluorescent specimens were mounted using Vectashield mounting media (Vector Laboratories, Burlingame, CA). Digital images (1,024 × 1,024 pixels) were acquired using a Zeiss LSM 510 Meta confocal microscope (Carl Zeiss Co. Ltd., Germany) and were imported into Photoshop (Adobe Systems, San Jose, CA). We used Photoshop software to decrease the background on confocal images with DAPI staining, and adjusted contrast of the DIC images to improve visualization of the cell morphology. Next, the cells were treated with OSM (20 ng/mL) for 48 h with or without pretreatment with stattic (1 μM for 1 h) for indirect immunofluorescence staining. The next steps were the same as those described above.

### Migration assay

Cell wounding assays were also conducted as described by Jones et al. [16], with minor modifications. Briefly, 5 × 10^5^ HTR8/SVneo cells were plated in 6-well plates in 2 mL medium. The cells were then incubated in a humidified chamber with 5% CO_2_ at 37°C until they reached confluence, and were then wounded using a sterile pipette tip, leaving a denuded area and a sharp demarcation line. Monolayers were then rinsed 4 times with s-PBS to remove the scraped cells. The cells were incubated for 12 h at 37°C in 5% CO_2_ with or without OSM (20 ng/mL) or function-blocking anti-gp130 antibodies (10 μg/mL; R&D system), and then photographed. Wound closure was assessed using a LEICA DM IRB/DC 300 microscope (Germany) at 100× magnification. Cell migration distance was measured using Olympus 6.51 software and compared with baseline measurements. To evaluate the effects of stattic (1 μM) on OSM-induced cell migrations, cells were incubated for 12 h at 37°C in 5% CO_2_ with or without OSM (20 ng/mL) or stattic (1 μM) and then photographed. The migration assay was performed as described above. Experiments were repeated at least 3 times in duplicate.

### Proliferation assay

HTR8/SVneo cells (1 × 10^4^) were plated in 96-well plate in a final volume of 100 μl/well culture medium in the absence or presence of OSM and stattic. Cells were incubated for 12 h and 48 h. After adding 10 μl of water-soluble tetrazolium (WST) reagent (Biovision, CA, USA) to each well, cells were incubated for 4 h in standard culture conditions. The absorbance of the samples was measured using a 96-well plate reader at 450 nm. The reference wavelength was 650 nm. Experiments were repeated at least 3 times in duplicate.

### Statistical analysis

Data are expressed as mean ± SEM. The non-parametric Mann–Whitney rank-sum test and an independent *t*-test were used to compare the 2 groups. A p-value of 0.05 or less was considered to be statistically significant. Each experiment was performed 3 times.

## Results

### Effects of OSM on mRNA and protein expression of E-cadherin in HTR8/SVneo cells

**Figure 1:**
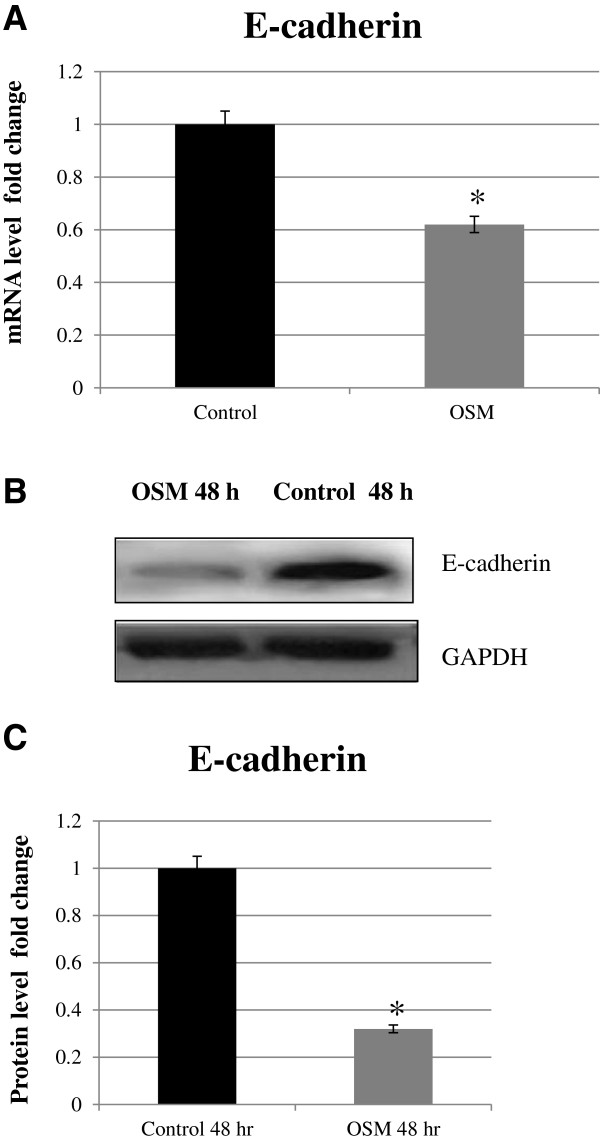
**Effects of OSM (20 ng/mL) on mRNA and protein expression of E-cadherin in extravillous cytotrophoblast cells (HTR8/SVneo).** Total RNA was extracted from the cells, with or without OSM stimulation for 48 h, and mRNA levels were measured by real-time quantitative RT-PCR analysis **(A)**. E-cadherin mRNA levels were standardized to GAPDH mRNA levels. Results are presented as means ± SEM of 3 independent experiments. The representative effects of OSM on E-cadherin as observed from the western blots are shown in relation to the control (48 h) **(B)**. Quantitative results **(C)** of western blots were normalized to GAPDH and presented as a fold change over the controls (untreated = 1). Data shown are the means ± SEM of 3 independent experiments compared to the control (*p < 0.05).

OSM (20 ng/mL) significantly reduced E-cadherin RNA and protein expression, compared to the control group (p < 0.05, respectively), after 48 h stimulation (Figure 1).

#### STAT3 phosphorylation is stimulated by OSM in HTR8/SVneo cells

**Figure 2:**
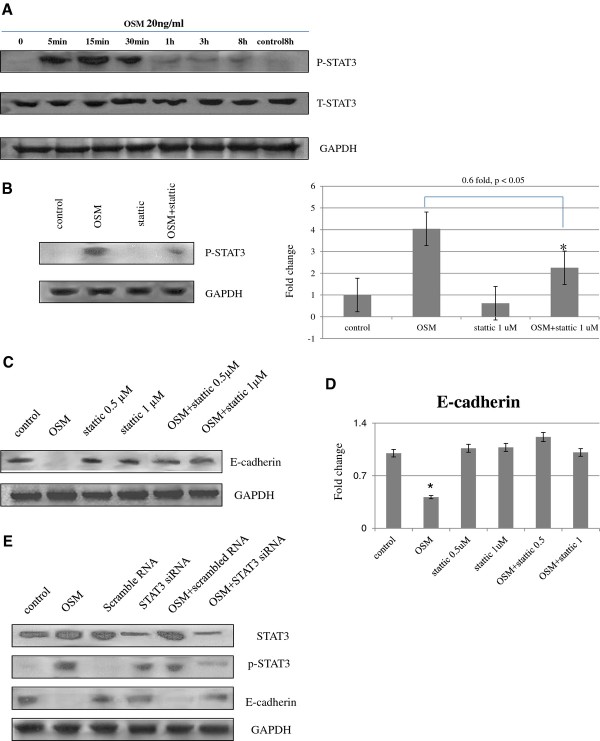
**Effects of OSM, STAT3 inhibitor, and STAT3 siRNA on protein expression of STAT3, phosphorylated STAT3 and E-cadherin.** STAT3 phosphorylation in HTR8/SVneo cells was transiently stimulated with OSM **(A)**. The effect of OSM on STAT3 phosphorylation was checked by pretreatment with stattic (1 μM for 1 h) compared to the OSM treatment, *p < 0.05 **(B)**. Cells were pretreated with the STAT3 inhibitor stattic (0.5 and 1 μM) for 1 h prior to treatment with or without OSM (20 ng/mL). Protein expression of E-cadherin was analyzed by western blotting **(C)**. The expression was quantified, and the values shown are means ± SEM **(D)** compared to the control, *p < 0.05. Western blots of STAT3, phosphorylated STAT3, and E-cadherin analyzed 24 h after transfection **(E)**. STAT3 and phosphorylated STAT3 protein expressions were reduced significantly after siRNA transfection compared with non-transfected control cells (p < 0.05, p < 0.05, 63.6% and 74.3%, respectively), whereas transfection with a non-targeted siRNA had no effect on STAT3 protein expression levels. Western blots of E-cadherin protein show significantly restored E-cadherin expressions (p < 0.05, 75.6%), compared to the OSM-induced E-cadherin suppression without affecting GAPDH protein expression. Non-targeted negative control siRNA did not affect OSM-induced E-cadherin suppression. The results are representative of 3 independent experiments.

Basal levels of STAT3 phosphorylation were very low, although stimulation with OSM led to immediate and transient increases in phosphorylation (Figure 2A). Total STAT3 protein expression did not change significantly at any time point. Stattic (1 μM for 1 h), a STAT3 inhibitor, suppressed OSM-induced STAT3 phosphorylation (0.6-fold, p < 0.05) in HTR8/SVneo cells (Figure 2B).

#### Effect of stattic on OSM-mediated changes in E-cadherin expression in HTR8/SVneo cells

To investigate the role of the STAT3 pathway in the OSM-induced downregulation of E-cadherin, HTR8/SVneo cells were pretreated with stattic (0.5 or 1 μM), which has been reported to inhibit the phosphorylation of STAT3, and then stimulated with OSM (20 ng/mL, 48 h). In western blotting, the expression of E-cadherin, which was suppressed by OSM, at 48 h, was restored by stattic pretreatment regardless of the concentration used (Figure 2C and D).

#### Effect of STAT3 siRNA on OSM-mediated changes in E-cadherin expression in HTR8/SVneo cells

Applying the described siRNA method and oligonucleotide sequence, the cellular contents of STAT3 and phosphorylated STAT3 were significantly decreased (p < 0.05, 63.6% and 74.3%, respectively) in HTR8/SVneo cells when 25 nM relevant oligos, but not when scrambled oligos were used, as analyzed by western blotting (Figure 2E). Transfection of HTR8/SVneo cells with STAT3 siRNA significantly increased E-cadherin expression (p < 0.05, 75.6%) which was suppressed by OSM without affecting the expression of the GAPDH protein. Non-targeted negative control siRNA did not affect the expression of STAT3 and E-cadherin expression.

### Effects of OSM and STAT3 inhibitor on E-cadherin in HTR8/SVneo cells by indirect immunofluorescence staining

**Figure 3:**
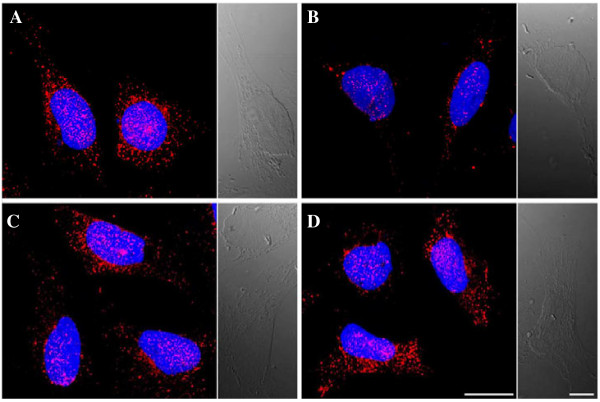
**Indirect immunofluorescence staining of E-cadherin.** Compared with unstimulated cells **(A)**, incubation of HTR8/SVneo cells in the presence of 20 ng/mL OSM for 48 h resulted in a robust downregulation of E-cadherin **(B)**, whereas there was no significant expression change after pretreatment with stattic (1 μM, 1 h) **(C)**. The expression of E-cadherin after pretreatment with stattic and 48 h incubation with OSM was similar to the expression of E-cadherin in unstimulated cells **(D)**. Results shown are representative of 3 independent experiments. Magnification, 1000×. Cells were counterstained with DAPI. Scale bar, 20 μm; differential interference contrast scale bars, 10 μm.

After 48 h of incubation in the presence of OSM, HTR8/SVneo cell staining revealed a downregulation of E-cadherin compared with the controls (Figure 3A and B). There was no specific change in the expression of E-cadherin, with or without stattic pretreatment (Figure 3A and C). E-cadherin expression after pretreatment with stattic and after 48 h incubation with OSM was similar to the expression in unstimulated cells (Figure 3A and D).

### Effects of OSM and STAT3 inhibitor on *in vitro* trophoblast migration

**Figure 4:**
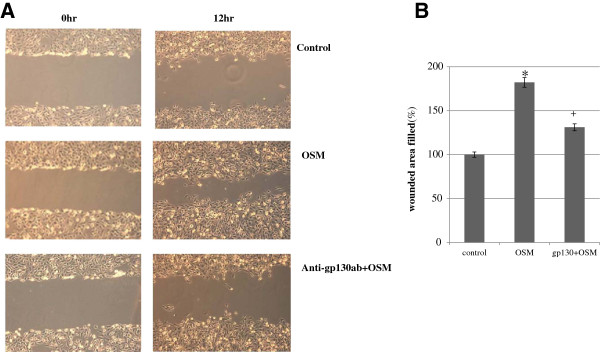
**HTR8/SVneo cell migration assessed by cell wounding assay.** The effect of OSM (20 ng/mL) with or without anti-gp130 antibodies (10 μg/mL) on the migration distance of cells after 12 h of culture **(A)**. Cell migration distance was measured using Olympus 6.51 software and compared with baseline measurements. Three independent experiments were performed. Numerical data were evaluated statistically and are presented as a histogram **(B)**. Data are expressed as the percentage of untreated control values given as mean ± SEM, with significant differences at *p < 0.01 for OSM and ^+^p < 0.05 for OSM and anti-gp130 antibodies, compared to the control. A representative result from 3 experiments is shown in **(A)**.

OSM induced a significant increase in cell migration distance—182.2% (p < 0.01) of the control—after 12 h of culture (Figure 4). Numerical data were evaluated statistically and are presented in the histogram shown in Figure 4B. When the anti-gp130 antibody (10 μg/mL) was used to treat the cells, the migration distance increased to 131.1% of the control (p < 0.05).

#### Relevance of the STAT3 signaling pathway in the OSM-mediated migration of HTR8/SVneo cells

**Figure 5:**
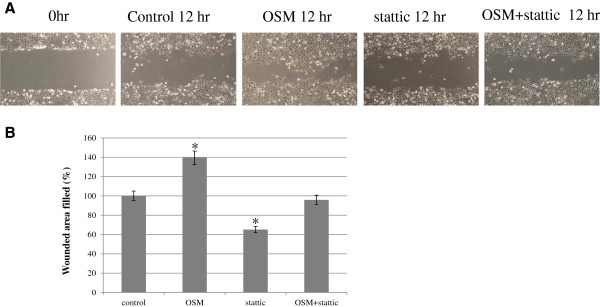
**Effects of STAT3 inhibition on OSM-induced migrations in HTR8/SVneo cells.** Cells were incubated for 12 h at 37°C in 5% CO_2_ with or without OSM (20 ng/mL), or with or without pretreatment with stattic (1 μM) for 1 h. Cell wounding assays were conducted and assessed using an LEICA DM IRB/DC 300 microscope (Germany) at 100× magnification. Cell migration distance was measured using Olympus 6.51 software and compared with baseline measurements. Three independent experiments were performed. A representative experiment from these experiments is shown in **(A)**. Results were expressed as a percentage of the control (means ± SEM) **(B)** compared to the control, *p < 0.05.

Stattic was used to investigate the relevance of STAT3-associated signaling in the OSM-mediated migration of HTR8/SVneo cells. Treatment of cells with a non-cytotoxic concentration of stattic (1 μM) resulted in a significant decrease (65.1%, p < 0.05) in migration compared with the vehicle control (Figure 5). Furthermore, when cells were co-treated with stattic and OSM, significantly increased migration by OSM 139.9%, p < 0.05) became not significant, compared with the control.

### Effects of OSM and STAT3 inhibitor on *in vitro* trophoblast proliferation

**Figure 6:**
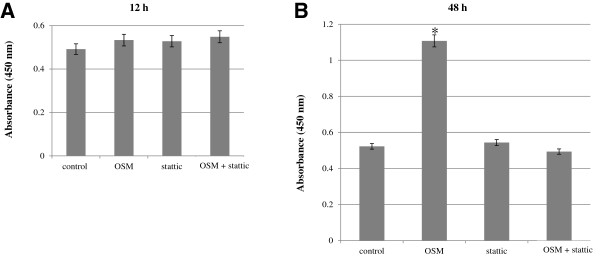
**The effects of OSM and STAT3 inhibitors on *in vitro *trophoblast proliferation.** OSM (20 ng/mL) did not induce significant increase in cell proliferation after 24 h of culture **(A)**, but induced a significant increase in cell proliferation after 48 h of culture to 2.1-fold (*p < 0.01) of the control **(B)**. Cell proliferation was significantly decreased with stattic (1 μM) treatment, compared to the control (^+^p < 0.05), at 48 h experiment **(B)**, not at 12 h experiment **(A)**. There was no significant difference in cell proliferation between the results of the co-treatment of OSM with stattic and the control **(A** and **B)**. Data shown are means ± SEM of 3 independent experiments. The absorbance of samples was measured using a 96-well plate reader at 450 nm. The reference wavelength was 650 nm.

OSM induced a significant increase in cell proliferation—2.1 fold (p < 0.01) of the control—after 48 h of culture, although OSM did not induce a significant increase after 12 h of culture (Figure 6). Numerical data were evaluated statistically and are presented in a histogram. Cells were co-treated with stattic (1 μM) and OSM to investigate the relevance of STAT3-associated signaling in OSM-induced proliferation. A significant decrease (p < 0.05) in proliferation was observed compared with cells treated with OSM alone, at the 48 h experiment (Figure 6B).

## Discussion

Tissues normally consist of epithelial or mesenchymal cells. Epithelial cells may be induced to change to a mesenchymal phenotype through EMT, an organized process first recognized in developmental biology as a means of achieving morphogenetic changes. In the instances where EMT is not controlled, pathologies arise whereby cell growth, proliferation, migration, and invasion are altered. A key example of this is carcinoma progression, whereby cells, which normally show resting epithelial morphologies, acquire a mesenchymal migratory potential and translocate to distant sites before reverting to an epithelial phenotype [17,18]. The expression of epithelial markers is reduced, while mesenchymal marker expression is increased. OSM has been identified as an EMT factor in lung and pancreatic tumor models [19]. It has also recently been reported that oncostatin-M promotes EMT, including E-cadherin loss in breast cancer [20]. In human renal tubular cells, it has been shown that OSM induces EMT through the Jak/Stat pathway and ERK signaling [21,22].

E-cadherin is usually expressed in epithelial cells and is involved in calcium-dependent cell-cell adhesion. In the placenta, E-cadherin mediates a strong intercellular interaction between adjacent trophoblast cells. During the first trimester of pregnancy, trophoblastic E-cadherin expression is temporarily down-regulated so that the EVTs acquire invasiveness [23]. Recent studies support the important role of E-cadherin in the regulation of the invasive behavior of human trophoblast cells [10,24].

In the present study, we used real-time PCR analysis, western blotting, and indirect immunofluorescence staining to demonstrate that the expression of the epithelial marker E-cadherin was significantly decreased by OSM. We also demonstrated that OSM stimulated the migration of HTR8/SVneo cells and that the addition of an anti-gp130 antibody decreased the stimulatory effects of OSM on migration. OSM belongs to the IL-6 family of cytokines and acts on target cells by binding to a heterodimeric membrane receptor composed of LIF- or OSM-specific receptor and the gp130 receptor chain [25]. In addition, OSM stimulated the proliferation of HTR8/SVneo cells at 48 h assay, not at 12 h assay. It is considered that significant increase in cell migration distance by OSM (12 h) represents an increased migration by OSM, because proliferation has not been changed significantly at 12 h assay. It has been shown that phosphorylated STAT3 enhances the invasiveness of tumors and trophoblast cells, where it is mainly activated by LIF [26]. We demonstrated that the migration and proliferation of trophoblasts are stimulated, E-cadherin is suppressed by OSM, and that these events are related to STAT3 phosphorylation. The down-regulation of E-cadherin by OSM was restored following treatment with a STAT3 inhibitor. In addition, OSM-stimulated migration and proliferation were significantly suppressed by STAT3 inhibition. Because it has been recently reported that a STAT3 inhibitor, stattic, has limitations to inhibit STAT3, selectively [27], we investigated the STAT3 pathway with STAT3 siRNA. The down-regulation of E-cadherin by OSM was restored following treatment with a STAT3 siRNA, with the same pattern. These results suggest that OSM stimulates the migration and proliferation of trophoblasts through STAT3 signaling, although the other pathway could be engaged by OSM, with or without STAT3 signaling.

No data regarding the effects of OSM on EMT in EVTs have yet been published. It has been reported that a significantly higher expression of OSM was identified in the cytotrophoblasts, syncytotorophoblasts and endothelium of the preeclamptic placenta compared with the normal placenta [8]. On the basis of the present study, OSM was found to induce the migration and proliferation of EVTs, through the down-regulation of E-cadherin. The effects of OSM on E-cadherin observed and the migration and proliferation of EVTs were contrary to observations that the invasion of EVT is shallow and that expression of OSM is elevated in the preeclamptic placenta [10,28-30]. The elevated expression of OSM in the preeclamptic placenta could be an adaptive phenomenon to rescue the shallow invasion of EVT. Another possibility is that the increased expression of OSM in preeclampsia may not be related to the effects of OSM on migration, proliferation, and invasion of EVTs, but instead could be related to the other effects of OSM. However, we do not yet know the effects of OSM on trophoblast migration, proliferation, and the invasion of EVTs in hypoxic environments. Recently, it was reported that recombinant interleukin-6 (IL-6) and TNFα were capable of activating endothelial cells, which is a hallmark of preeclampsia [31]. Another study demonstrated that IL-6 stimulates cell migration and invasion accompanied by the increased expression of related integrin subunits on the HTR8/SVneo cell line [32], although the former study only suggested the effects of IL-6 on EVT invasion cellular cascades [33]. LIF, a member of the IL-6 family, has been suggested to increase the invasiveness of trophoblastic cells through the activation of STAT1 or STAT3 [26,34]. Because OSM is a cytokine in the IL-6 family, its role in activating endothelial cells should be investigated to evaluate the role of OSM in the preeclamptic placenta [31,32]. The functional role of OSM in the human placenta has not yet been clarified. Because OSM has cell-type specific effects, the effects and mechanisms of OSM related to normal and pathologic pregnancies should be evaluated both *in vitro* and *in vivo*.

## Conclusions

Taken together, these data suggest a contributing role for OSM in stimulating the migration of EVTs during the first trimester through down-regulation of E-cadherin. The effects of OSM on E-cadherin and the migration of the trophoblasts were related to STAT3 activation, which is important for trophoblast invasiveness. Further research is needed to investigate the various roles of OSM in normal and pathologic pregnancies under hypoxic conditions, including how this cytokine interacts with other regulating molecules.

## Competing interests

The authors declare that they have no competing interests.

## Authors’ information

Hyun Sun Ko: Clinical Assistant professor of the Catholic University of Korea, Division of Maternal-fetal Medicine, Department of Obstetrics and Gynecology, Seoul St. Mary’s Hospital of the Catholic University of Korea. Sae Kyung Choi: Clinical Assistant professor of the Catholic University of Korea, Division of Maternal-fetal Medicine, Department of Obstetrics and Gynecology, Seoul St. Mary’s Hospital of the Catholic University of Korea

Ho Shik Kim: Professor of the Catholic University of Korea, Department of Biochemistry, College of Medicine, Catholic University of Korea. In Yang Park: Associated Professor of the Catholic University of Korea, Division of Maternal-fetal Medicine, Department of Obstetrics and Gynecology, Seoul St. Mary’s Hospital.

Jong Chul Shin: Professor of the Catholic University of Korea, Division of Maternal-fetal Medicine, Director, Department of Obstetrics and Gynecology, Seoul St. Mary’s hospital, President of the Korean Society of Maternal Fetal Medicine.
